# Generation and characterization of D-dimer specific monoclonal antibodies for use in latex agglutination test

**DOI:** 10.1371/journal.pone.0212104

**Published:** 2019-02-14

**Authors:** Beáta Török-Nagy, József Antal, Béla Dénes

**Affiliations:** 1 Doctoral School, University of Veterinary Medicine, Budapest, Hungary; 2 Department of Coagulation, Diagon Ltd., Budapest, Hungary; 3 Laboratory of Immunology, Veterinary Diagnostic Directorate of the National Food Chain Safety Office, Budapest, Hungary; Duke University School of Medicine, UNITED STATES

## Abstract

The commercially available D-dimer assays used in the clinical practice often show differences in the results, and their specificity and sensitivity are rather unsatisfactory. Our aim was to develop a new monoclonal antibody against D-dimer with a proper specificity, and estimating its suitability using in a latex agglutination diagnostic test. Monoclonal antibodies were generated using hybridoma technology. Their titer was determined by a self-developed ELISA method. The cross-reactions of the antibodies were tested. Characterization of the epitope specificity of a selected antibody was performed through digestion of D-dimer followed by Western blotting. The amino acid sequences of the active antigen fragments were determined. According to the ELISA results, 38 cell groups were constated as antibody-producing hybridomas, among them 7 gave raised titer of antibody and were cloned. Based on the cross-reaction analysis, none of the antibodies gave cross-reaction with fibrin-E and fibrinogen-E fragments but reacted with fibrin D and fibrinogen D fragments. A low cross-reaction was showed with fibrinogen and fibrin X and Y. Contrary to the others, antibody 2B9 gave no cross-reaction with fibrinogen and reacted weakly with fibrin X and Y fragments. According to the epitope analysis the antibody 2B9 binds to amino acids 94–99 and to amino acids 140–147 on the beta chain and it recognizes the amino acids 23–32 and 93–98 on the gamma chain of D-dimer. Considering the characteristics of the above mentioned monoclonal antibody 2B9, we found that it is suitable to be a basis for a D-dimer diagnostic test with proper specificity.

## Introduction

The D-dimer test plays a very important role in diagnosis and monitoring of thrombosis and other diseases impacting blood coagulation in human and veterinary medicine. Primarily, it has an overriding importance in the exclusion of venous thromboembolism (VTE), in particular deep vein thrombosis and pulmonary embolism.

A positive test result indicates an elevated D-dimer level in blood, which may be caused by secondary fibrinolysis, but also by trauma, pregnancy, sepsis, inflammation or other factors, therefore the positive result does not necessarily mean the presence of VTE [1]. The age can also correlate with D-dimer levels, so elderly people may have higher D-dimer levels. [2–3] However, a negative result practically should exclude the presence of deep vein thrombosis and pulmonary embolism with high certainty, in case it coexists with low or intermediate pretest probability based on clinical score systems (Wells and Geneva models) [4–6]. The commercially available D-dimer assays used in the clinical practice often show differences in the results [7–8], and their specificity and sensitivity are rather unsatisfactory [9]. This may be caused by different reasons, mainly the specificity of the antibody [10], cross-reactions with degradation products of fibrinogen, which can result in different measured D-dimer values. Several tests utilize one monoclonal antibody only, while in others two are applied [11,1]. Globally, the standardization of the different tests and a D-dimer standard to which the several tests should be calibrated should be required [12–13].

The importance of measurement of D-dimer in diagnostic use underlines the requirement of availabity of highly specific D-dimer tests. This was the reason for development of a new D-dimer specific monoclonal antibody. This paper is intended to present the process of this development and the characterization of the resulted monoclonal antibody as well as the preparation of a D-dimer antigen, generation of a panel of monoclonal antibodies through immunization of mice and by hybridoma and cloning techniques. Thereafter antibodies were continously produced by hybridoma cells. Generated antibodies were selected with immunological characterization and the most promising candidate regarding its potential in diagnostic applications was characterised.

## Materials and methods

### Preparation of the D-dimer antigen

The D-dimer antigen was prepared through the digestion of fibrin. To generate a fibrin mass, thrombin time reagent (Diagon Ltd. Budapest, Hungary) containing thrombin and calcium were added to human plasma and were incubated for 90 minutes at 37°C. The generated clot was centrifuged, then suspended and incubated at 37°C in TSC buffer (pH 7.4). After an overnight incubation, the clot was washed several times. The fibrin polymer was digested by plasmin (Calbiochem, San Diego, CA) in a final concentration of 2.5 μg /ml. The treatment was performed at 37°C, by shaking the samples continously. The supernatant was collected, and substituted to TSC buffer, then enzyme was added again to the fibrin mesh. These steps were repeated until the clot was totally digested. The collected lysate was purified on Sephadex G-25 column (GE Healthcare, Uppsala, Sweden) and then was concentrated with Amicon Ultra Centrifugal Filters (100,000 MWCO) (Merck Millipore, Billerica, MA). The generated degradation product was analysed with sodium dodecil sulphate polyacrylamide gel electroforesis (SDS-PAGE), its molecular weight was determined with Western blotting. The functional activity was measured with an ELISA method (Asserachrome D-dimer, Diagnostica Stago, Inc., Parsippany, NJ) and with a D-dimer latex immunoturbidimetric assay (DiaSys Diagnostic Systems GmbH, Holzheim, Germany). The prepared D-dimer antigen was used for mouse immunization.

### Immunization

Three 8-week-old female Balb/c AnN Crl BR mice (Charles River, Wilmington, MA) were immunized intraperitoneally (i.p.) and three other subcutaneously (s.c.) with 200 μl of a mixture of the D-dimer antigen and complete Freund’s adjuvant (CFA) (Sigma Aldrich Co., St. Louis, MO). After 24 days, the mice were injected in the same manner (i.p. or s.c.), using incomplete Freund’s adjuvant (IFA) (Sigma Aldrich Co., St. Louis, MO). Ten days after, blood samples were collected from each mouse to define the level of produced antibody with a self-developed indirect ELISA method. Four weeks atfer the last immunization one mouse with the highest antibody response was inoculated intravenously (i.v.) with the 50 μl of D-dimer antigen diluted 1:2 in physiological saline. All animals were handled in strict accordance with the recommendations in the Guide for the Care and Use of Laboratory Animals of the Veterinary Diagnostic Directorate of the National Food Chain Safety Office. The animal use protocol was approved by the Institutional Animal Care and Use Committee.

### Cell fusion

Three days later the mouse was sacrified, and its spleen was removed aseptically. The fusion of spleen cells with Sp2/0-Ag14 murine myeloma cells was performed in the presence of polyethylene glycol (Sigma Aldrich Co., St. Louis, MO). The cell mixture was measured into 96-well microtiter plates precultivated with murine peritoneal macrophages. The fused cells were allowed to grow in a carbon dioxid gas incubator, containing 5% CO2 at 37°C.

The obtained hybrid cells were selected with hypoxanthine, aminopterine, and thymidine (HAT) medium (Sigma-Aldrich Co.). One week later the HAT medium was substitued for an HT medium. Following 2 weeks of cultivation the hybridoma cells were screened by testing the presence of antibodies in the culture supernatants by the indirect ELISA method.

### Testing of cell supernatants by indirect ELISA

The wells of the ELISA microtiter plates (Analyzer Ltd., Budapest, Hungary) were coated with D-dimer antigen, diluted in carbonate buffer (pH = 9.6). 100 μl aliquots were measured into the wells of the plates), and allowed to incubate at 4°C overnight. Then the plates were washed three times with washing-diluting PBS buffer, containing 0.05% Tween 20 (Diavet Ltd., Budapest, Hungary). Thereafter the cell culture supernatant, diluted 10-fold with washing-diluting buffer was added to each well, in a volume of 100 μl. RPMI media (Sigma-Aldrich Co.) supplemented with 10% fetal calf serum (Sigma-Aldrich Co.), was used as a negative control. After one hour of incubation at 37°C, the wells were washed three times. One-hundred μl of a peroxidase-conjugated goat anti-mouse IgG (H+L) (Jackson Immuno Research Labs Inc., West Grove, PA) diluted 2,500-fold with PBS-Tween-20 buffer, was added to wells. After 1 hour of incubation and washing procedure, 100 μl of tetramethylbenzidine substrate (TMB) (Diavet Ltd., Budapest, Hungary) was mesured into the wells, and allowed to stand for 6 minutes. The colour reaction was stopped with 50 μl of 4 N H2SO4 solution. Then the absorbance was mesured at 450 nm using a FLUOstar Optima (Thermo Fisher Scientific, Waltham, MA USA) microplate reader.

### Cloning, purification of monoclonal antibodies (mAbs)

Seven cell groups were cloned from 2 to 5 times by end-point dilution method and were cultivated in flasks at 37°C in a carbon dioxide (5%) incubator. The supernatants of the grown cells were tested with the self-developed indirect ELISA, and in parallel with microscopical examination. The hybridomas were cultured in flask culture and in miniPERM bioreactor (Sarstedt, Nümbrecht, Germany) using RPMI-1640 medium supplemented with 10% fetal bovine serum, antibiotic antimycotic solution (all from Sigma-Aldrich Co). Cell supernatants were continuously collected and then were purified and concentrated by Protein G- Sepharose column (GE Healthcare, Uppsala, Sweden).

### Study of cross-reactions

The possible cross-reactions of the produced antibodies were examined by the self-developed indirect ELISA. Different fibrin degradation products, exactly fibrin-D monomer, fibrin-E monomer, fibrin-X fragment, fibrin-Y fragment, and also fibrinogen degradation products, fibrinogen-D monomer, fibrinogen-E monomer and fibrinogen were used as antigens (all from BIOTREND Chemikalien GmbH, Cologne, Germany). The antigens were diluted with carbonate buffer (pH = 9.6), in 2.5μg/ml working concentration. The ELISA was performed as described above. Regarding the results, the intensity of the examined cross-reactions was expressed with the symbol (+) by calculating the following ratio of the measured optical densities: OD _cell supernatant_ / OD _negative control_. The defined categories of the intensity are the following: ratio < 2 no reaction (-); 2 < ratio> 3 weak cross-reaction (+); 3 < ratio> 5 modetare cross-reaction (++); 5 < ratio> 8 strong cross-reaction (+++); ratio > 8 very strong reaction (++++).

### Determination of immunoglobulin class

The isotype based on to the heavy and light chains of the produced antibodies was determined using a Mouse Monoclonal Antibody Isotyping Test Kit (AbDSerotec, Raleigh, NC).

### Epitope mapping (SDS-PAGE, Western blotting)

Digested antigen fragments were tested with the produced monoclonal antibodies by Western blotting, in order to identify the epitope of the antigen.

The D-dimer antigen applied during the immunization was dissolved in 70% formic acid and was incubated with cyanogen bromide (CNBr) (all from Sigma Aldrich Co.). The CNBr cleavage was performed at 25°C, in darkness, under argon for 24 hours. The reaction was stopped by adding destilled water to the reaction mixture. Residual cyanogen bromide was removed with lyophilization. After lyophilization, fragments were dissolved in PBS buffer and were analyzed by SDS- PAGE to determine their molecular weight. The CNBr derived fragments were digested further by chymotrypsin (Sigma Aldrich Co.). The chymotrypsin digested products were also analyzed by 4–20% SDS-PAGE. The digested antigen fragments were treated with Laemmli buffer (Bio-Rad Laboratories Hercules, CA), and were boiled for 5 minutes. Previously the buffer was supplemented with 5% 2-mercaptoethanol (in the case of the reduced samples). The samples were mesured into the wells of a Clear PAGE SDS Gel 4–20% (C.B.S. Scientific Co., Inc., Del Mar, CA) and were electrophoretised at 110 V for 1.5 h using the Mini-Vertical Slab Gel/Blotting System DCX-700 (C.B.S. Scientific Co. Inc.). After electrophoresis, proteins were transferred onto a PVDF membrane (Merck Millipore, Billerica, MA) using an ECL Semi-dry Blotter (Amersham Biosciences). To avoid aspecific bindings the membrane was treated overnight in PBS blocking solution. Then monoclonal antibody 2B9 was added to the solution, and the membrane was incubated for 2 hours at room temperature. After the washing steps in PBST buffer (PBS; 0.05% Tween-20) the membrane was incubated for 1 hour with goat anti-mouse IgG (H+L)-HRP conjugate (Jackson Immuno Research Labs) diluted 2500 folds with PBS. The proteins were blotted to PVDF membrane and among them the active fragments–reacting with the produced D-dimer specific monoclonal antibody 2B9 –were visualized by Amersham ECL Western Blotting Detection Reagents (GE Healthcare, Uppsala, Sweden) and subsequent exposure to Amersham Hyperfilm ECL (GE Healthcare, Uppsala, Sweden). After identifying the bands of interest, they were cut out from the membrane, and were sequenced based on the Edman degradation protocol [14] using an automated ABI 494 protein sequencer (Applied Biosystems, Foster City, CA, USA).

## Results

To the generation of D-dimer-specific monoclonal antibodies, a D-dimer antigen was prepared from human plasma, via producing a fibrin clot then digesting it with human plasmin. The collected lysate was purified on Sephadex G-25 column (GE Healthcare, Uppsala, Sweden). The purity of the generated degradation product was analyzed with sodium dodecil sulphate polyacrylamide gel electroforesis performed under non-reducing conditions. The molecular weight of D-dimer was determined with immunoblotting, resulting 180 kDa.

The functional activity and the concentration of D-dimer antigen was mesured by a D-dimer latex immunoturbidimetric assay (DiaSys, Holzheim, Germany), it resulted 2 mg/ ml.

The analysed and purified D-dimer antigen was used to immunize BALB/c mice, and hybridoma cells were generated by fusing the spleenocytes of mice with pre-cultivated Sp2 murine myeloma cells. After the fusion, hybrid cells were selected in a specific media, and consequently we obtained 576 hybridoma cell groups. The antibodies produced by the hybridomas were screened by the self-developed indirect ELISA using D-dimer antigen. According to the results of ELISA, 38 D-dimer-positive hybridomas cell groups were found, producing D-dimer specific antibodies. Among them 7 cell groups (designated 1B4, 1H12, 2B9, 2F2, 3B3, 4G8, 6B9) were selected regarding their titer of produced antibody and were subjected to several cloning steps by limiting dilution.

According to the isotype determination of antibodies, 2F2 had IgM isotype antibody, and all other tested mAbs 1B4, 1H12, 2B9, 3B3, 4G8, 6B9 had IgG isotype belonging to IgG1 subclass regarding the heavy chain and all of them had κ light chains.

During further characterization of the produced mAbs, the possible cross-reactions of the antibodies were tested with fibrinogen, fibrinogen degradation products (fibrinogen D monomer, fibrinogen E monomer) and with fibrin degradation fragments (Y, X, D, E) (all from BIOTREND Chemikalien GmbH). A commercially available Anti-D-dimer monoclonal antibody (DD3) (HyTest Ltd., Turku, Finland) was used as a reference antibody. (Table 1).

**Table 1:** Cross reactions of D-dimer specific monoclonal antibodies produced by different cell lines and tested by indirect ELISA.

Mabs	Native protein		Fragments					
	D-Dimer	Fibrinogen	Fibrinogen		Fibrin			
			D	E	D	E	X	Y
**1B4**	++++	++	+++	-	++	-	+++	+++
**1H12**	++++	++	+++	-	++	-	+++	+++
**2B9**	++++	-	+++	-	+++	-	+	++
**2F2**	+	++	+	-	+	-	+	+
**3B3**	++++	+++	+++	-	++	-	+++	+++
**4G8**	+++	+	++	-	+	-	+	++
**6B9/**	+++	++	++	-	+	-	-	++
**HyTest Anti-D-Dimer**^a^	+++	-	++++	-	++++	-	+	+++

Intensity of the reaction: ++++ very strong; +++ strong, ++ moderate; + weak; and—no reaction.

^a^Commercially available Anti-D-dimer monoclonal antibody (HyTest Ltd.) was used as reference.

According to the ELISA results, all analysed antibodies reacted with D-dimer, but the following mAbs showed a very strong reaction: 1B4, 1H12, 2B9, 3B3. These mAbs reacted also with fibrin D and fibrinogen D fragments but did not cross-react with fibrin-E and fibrinogen-E fragments. The antibodies produced by cell lines 2F2, 4G8 and 6B9 reacted weakly with D-dimer and gave no or just weak cross-reaction with D-fragments. With high molecular weight fragments, fibrin X and Y, the mAbs 1B4, 1H12 and 3B3 showed a strong cross-reaction, and with other mAbs just a low reaction was found. The cross-reaction with fibrinogen was mostly strong or moderate (3B3, 1B4, 1H12, 2F2) or weak (4G8, 6B9), however in the case of the mAbs 2B9, there was no reaction with fibrinogen. The results revealed that antibody designated 2B9 was quite similar to the reference HyTest Anti-D-dimer monoclonal antibody (HyTest Ltd.), both of them reacted strongly with D-dimer, but gave no cross-reaction with fibrinogen and reacted weakly with the fibrin X and Y fragments. Henceforth we focused on the antibody 2B9, and the surfaces it possibly recognizes on a D-dimer molecule were determined as follows.

**Fig 1:**
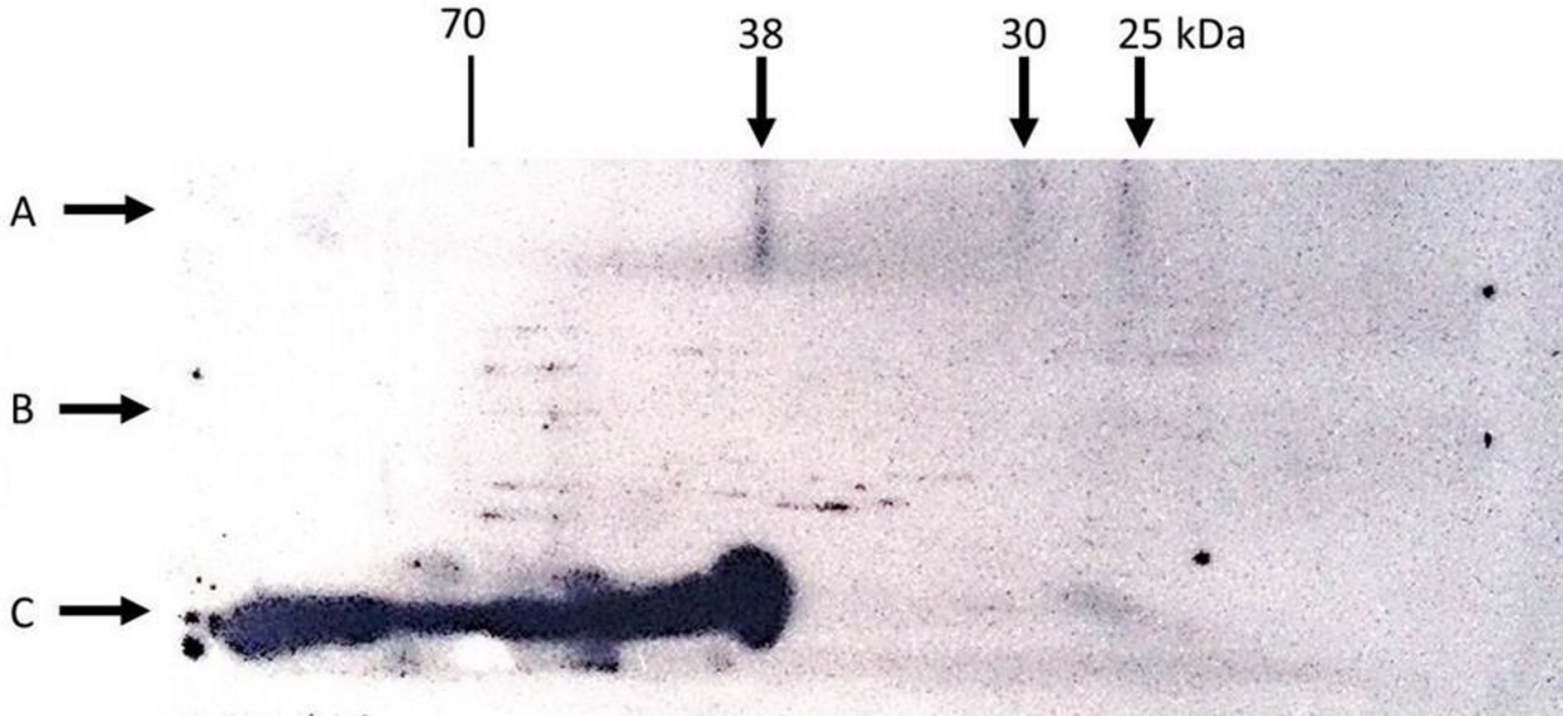
**Western blotting analysis of native (C) and digested D-dimer antigen under reducing (B) and non-reducing (A) conditions with a 2B9 monoclonal antibody**.

Identifying surfaces on the D-dimer molecule reacting with 2B9 antibody revealed a more spatial epitope structure than a simple sequential epitope. According to Fig 1, Western blots of reduced and unreduced forms of chymotryptic digest showed interaction with bands representing 25, 30 and 38 kDa fragments of the unreduced sample, while the non-disulfide bonded peptid chains—made by reduction of the mentioned fragments—showed no interaction with 2B9 antibody.

**Fig 2:**
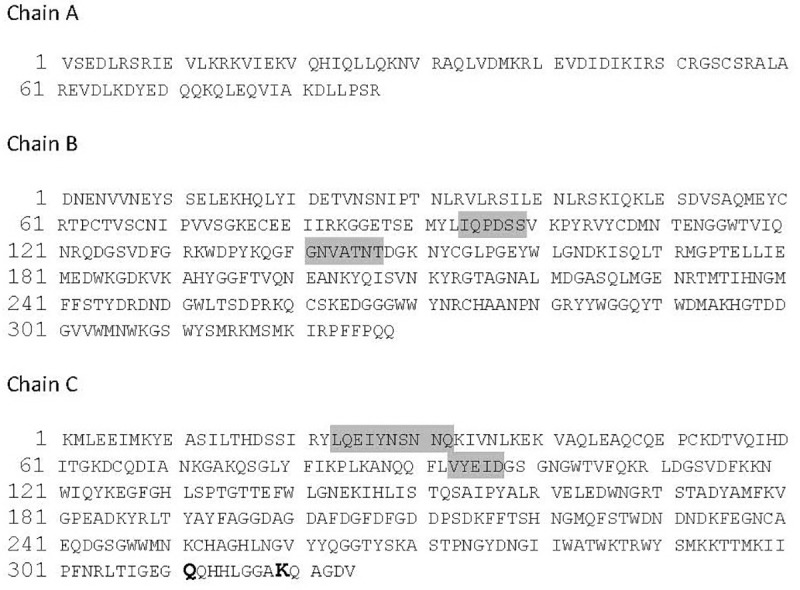
Chains, dimerization sites and identified fragments of fibrin D monomer. Highlights: gray–fragments identified by Edman sequencing. Dimerization residues are marked with bold characters.

After the separation and the immunological identification of the Mab targeted fragments of the unreduced digest, protein sequencing was accoplished in order to the narrow the range of possible epitope surfaces. The fragments identified with molecular weight of 25 and 38 kDa provided sequences whereas no sequences were obtained from the 30 kDa fragment probably due to N-terminal block. Since the identified N-termini were identical from the two signal-providing fragments, their different C-terminal digestion patterns should be supposed. As Fig 2 shows 94 IQPDSS- and 140 GNVANTNT- sequences were identified at chain B and 23 LQEIYNSNNQ- and 93 VYCEID- fragments were identified at chain C.

**Fig 3:**
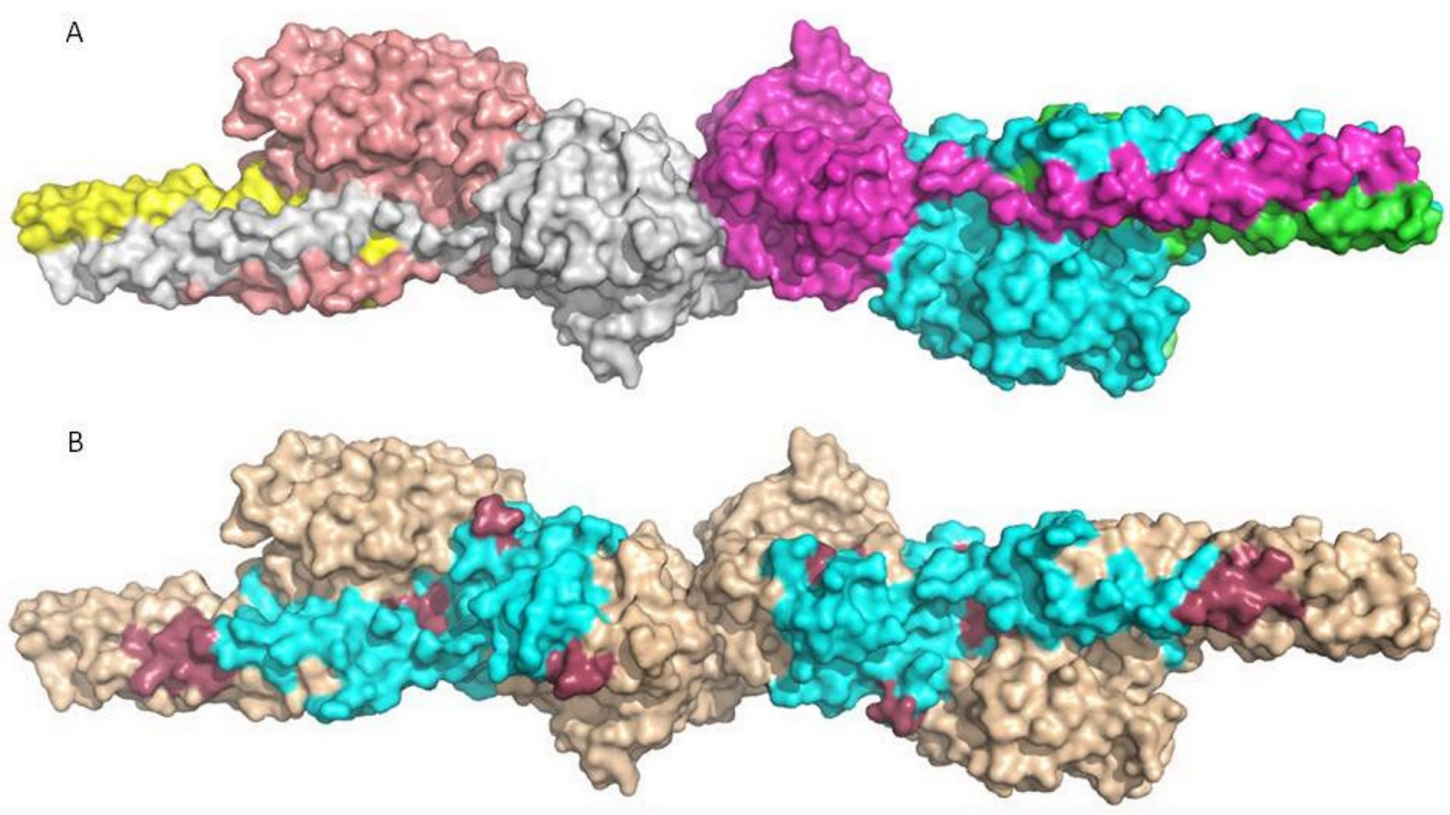
**Chains (A), epitope surfaces and identified digestion fragments (B) of fibrin D-dimer.** Highlights on A: yellow and green–chain A; rose and cian–chain B; silver and magenta–chain C. Highlights on B: beige–undefined surface, purple–fragments identified with peptide sequencing and cian–possible spatial epitope.

There were no sequences identified from Chain A, probably due to the fact that their N-termini was blocked during the digestion processes. The identified fragments might designate roughly half of the residues of Chain B and C as marked by blue coloration on the 3D structure, referring relatively wide ranges of possible interaction surfaces (Fig 3).

The marked epitope surfaces are located relatively distant on the two subunits, suggesting that antibody 2B9 could be an appropriate reporter molecule in latex immunoassays as steric interferences between two reacting antibodies have low probability. Since further endoproteinases with appropriate specificity to follow targeted digestions are not available, further narrowing of the epitope surfaces is difficult. Sophisticated limited digestions with exopeptidases followed by protein sequencing might provide deeper insight.

## Discussion

Our goal creating a higly specific D-dimer monoclonal antibody and estimating its applicability in a latex based immunoturbidimetric diagnostic assay was fulfilled. Regarding the mechanism of the turbidimetric D-dimer assay, that is polystyrene latex microspheres are coated with D-dimer specific monoclonal antibodies due to reaction with the D-dimer antigen of plasma samples are agglutinating, i.) the antibody has to specifically recognize the appropriate epitopes on the D-dimer molecules and ii.) the epitopes have to be located symmetrically, and far from each other in such a way that they could not cause any sterical blocking of agglutination of the latex particles.

Based on the results of cross-reaction analysis, we claim that monoclonal antibody 2B9 satisfied the criteria of high specificity, as it does not react with fibrinogen, which is an important characteristic of D-dimer specific antibodies using in D-dimer diagnostic tests.

Adam et al. studied the problems associated with D-dimer measurements and they found that one weakness of the D-dimer tests was their incapabability to distinguish fibrin degradation products and fibrinogen [11]. Consequently, when producing monoclonal antibodies, the detection of cross-reactivity is a key issue, it is required that the antibody should not react with fibrinogen. Our cross-reaction analysis resulted that out of the seven types of produced antibodies four reacted strongly or moderately, and two weakly with fibrinogen. Only antibody 2B9 gave no reaction with fibrinogen, thus it was considered as an antibody with adequate specificity to the D-dimer antigen.

This advantageous characteristic of novel antibody allows the use of citrated plasma or even whole blood- containing fibrinogen- in antibody-based assays [15].

The commercially available anti-D-dimer monoclonal antibodies detect the epitopes on the surface of the crosslinked fibrin fragment, D-dimer. Each of the monoclonal antibodies has its own specificity, and some of them have been epitope-mapped. The first patented anti-D-dimer monoclonal antibody (DD-3B6/22) was developed by Rylatt et al. [16]. According to the analysis of Wylie et al., the epitope surface identified by antibody DD-3B6/22 was located on the gamma polypeptide chain between the amino acids 86 and 88, and on the alpha polypeptide chain between the 105–110 amino acids [17–18]. Another D-dimer specific monoclonal antibody B4, discovered by Doh et al., binds to an antigen surface composed of amino acids 134 to 142 from the N-terminal region of beta polypeptide chain [19–20].

Our epitope analysis showed that the novel monoclonal antibody 2B9 recognizes other amino acid sequences on D-dimer, compared to the previous antibodies. It binds to amino acids 94–99 and to amino acids 140–147 on the beta chain and it recognizes the amino acids 23–32 and 93–98 on the gamma chain.

The epitope mapping proves that the produced antibody, designated 2B9, recognizes the epitopes situated symmetrically, and far from each other on the antigen surface. Their position could facilitate the agglutination of latex beads. We assume that our D-dimer specific monoclonal antibody is applicable to bind to the latex surface; thus it can be used in immunoturbidimetric assays based on agglutination. Our results indicate that the epitope residues are spatial on the antigen surface but we cannot clearly consider this type of epitope either advantageous or disadvantageous based on literature.

In certain cases, a spatial epitope could be preferable as it can also have diagnostic benefits. Comparing a diagnostic test using this antibody with other commercially available D-dimer tests–which contain an antibody recognising a sequential epitope–we assume that the antibody recognising spatial epitope could interact with antigen which is not a cross-linked fragment formed during the seconder fibrinolysis. With this test in combination with another one operating with appropriate sequential epitope, other types of diseases resulting in high D-dimer (or oligomer) level in plasma-which are not developed as a consequence of the thrombotic activity-, could also be diagnosed.

Our claim that the generated D-dimer specific monoclonal antibody 2B9 is suitable to use in a D-dimer latex agglutination diagnostic test was proven as anew D-dimer diagnostic test utilizing this antibody was developed and evaluated. Comparative measurements were made with this new test and another commercially available D-dimer test which resulted in good correlation, thus demonstrating the suitability of the new antibody for use in a diagnostic test [21].

## Supporting information

S1 File **Contains the following files: Appendix A**. Protocol of SDS-PAGE and Western blotting of D-dimer antigen. **Appendix B.** Protocol of mAbs production in bioreactor and purification. **Table A.** The composition of suspensions for immunization of Balb/c AnN Crl BR mice.
(DOC)

S1 Fig SDS-PAGE and Western blotting analysis of D-dimer antigen. A: SDS-PAGE (under non-reducing conditions) of D-dimer antigen. B: Western blotting analysis of D-dimer antigen with a commercially available anti-D-dimer monoclonal antibody (HyTest Ltd.). Mw: Molecular weight marker—SeeBlue Plus2 Pre-stained Protein Standard (Invitrogen).
(TIFF)
