# Supplementary material for: Generation and characterization of D-dimer specific monoclonal antibodies for use in latex agglutination test
Source: PLoS One. 2019 Feb 14;14(2):e0212104. doi: 10.1371/journal.pone.0212104 (PMC6375587; doi:10.1371/journal.pone.0212104)
Supplement: S1 File — Contains the following files: Appendix A. Protocol of SDS-PAGE and Western blotting of D-dimer antigen. Appendix B. Protocol of mAbs production in bioreactor and purification. Table A. The composition of suspensions for immunization of Balb/c AnN Crl BR mice. (DOC) [file pone.0212104.s001.doc]

Supporting Information S1 File

## **Appendix A. Protocol of SDS-PAGE and Western blotting of D-dimer antigen**

D-dimer antigen was treated with Laemmli buffer (Bio-Rad Laboratories, Hercules, CA) in a proportion 1:1, and was boiled for 5 min. to denature the proteins. The samples were measured in the wells of a 4–20*%* Mini*-*PROTEAN*®* TGX*™* Gel (Bio-Rad Laboratories, USA) and were electrophoretised at 150 V for 1.5h using the MiniVE electrophoresis system (Amersham Biosciences, Piscataway, NJ). The proteins were blotted to a nitrocellulose membrane using an ECL Semi- dry Blotter (Amersham Biosciences Co, Piscataway, NJ). To avoid aspecific bindings the membrane was treated overnight in PBS blocking solution, containing 3.5 % fat-free milk powder. Then the membrane was incubated for 2 hours at room temperature in a solution containing the anti-D-dimer monoclonal antibody (HyTest Ltd., Turku, Finland) diluted with PBS blocking buffer. After washing the membrane in PBST buffer (PBS; 0,05% Tween-20) it was incubated for 1 hour with a goat anti-mouse IgG (H+L)-HRP conjugate (Jackson Immuno Research Labs., Inc., West Grove, PA) diluted 2,500 folds with PBS. After repeating the washing steps described above, the membrane was stained with 3,3′-diaminobenzidine (DAB) substrate (Sigma Aldrich Co., St. Louis, MO) and 0.01% H2O2 was added to the solution. The antigen-antibody reaction was shown throught the chromogenic reaction, and the appropriate strips were stained.

## **Appendix B. Protocol of mAbs production in bioreactor and purification**

The continuous production of the D-dimer–specific monoclonal antibody was performed in flask culture and also in miniPERM bioreactor (Sarstedt, Nümbrecht, Germany). The hybridomas were cultivated in RPMI-1640 medium supplemented with 10% fetal bovine serum, antibiotic antimycotic solution (all from Sigma-Aldrich Co). To protect the cells against shear forces, cellPROTECT solution (Sarstedt, Nümbrecht, Germany) was added to the medium and to reduce the foam, antiFOAM (Sarstedt) was measured in the nutrient module. From the production module of the bioreactor the supernatant of hybridomas was harvested every second day, and centrifuged with 3000 g for 10 min. Cell supernatants were passed through a Protein G- Sepharose column (GE Healthcare, Uppsala, Sweden). The column was equilibrated with 20 mM phosphate buffer (pH 7.0), and antibody was eluted with 0.1 M glycine buffer (pH 2.7). The eluted antibody was neutralised with Tris buffer (pH=8) and then it was precipitated in the proportion of 1:1 with saturated ammonium-sulfate solution (pH=7,2), and stored at 4°C*.*

**~~Table A. The composition of suspensions for immunization of Balb/c AnN Crl BR mice.~~**

| **Mouse Nr.** | **Suspensions for immunization** | **i.p. / s.c.** |
| --- | --- | --- |
| 1. | 100 µl antigen + 100 µl CFA | i.p. |
| 2. | 100 µl antigen + 100 µl CFA | s.c. |
| 3. | 50 µl antigen + 50 µl PBS + 100 µl CFA | i.p. |
| 4. | 50 µl antigen + 50 µl PBS + 100 µl CFA | s.c. |
| 5. | 25 µl antigen + 25 µl PBS + 100 µl CFA | i.p. |
| 6. | 25 µl antigen + 25 µl PBS + 100 µl CFA | s.c. |

After 24 days, the mice were injected in the same manner (i.p. or s.c.) but the CFA was substituted for IFA.

Antigen: prepared D-dimer (1 mg/ml)

CFA: complete Freund’s adjuvant

PBS: phosphate buffered saline

IFA:incomplete Freund’s adjuvant

## i.p.: intraperitoneally

s.c.: subcutaneously
