# Supplementary figures and images for: Generation and characterization of D-dimer specific monoclonal antibodies for use in latex agglutination test
Source: PLoS One. 2019 Feb 14;14(2):e0212104. doi: 10.1371/journal.pone.0212104 (PMC6375587; doi:10.1371/journal.pone.0212104)

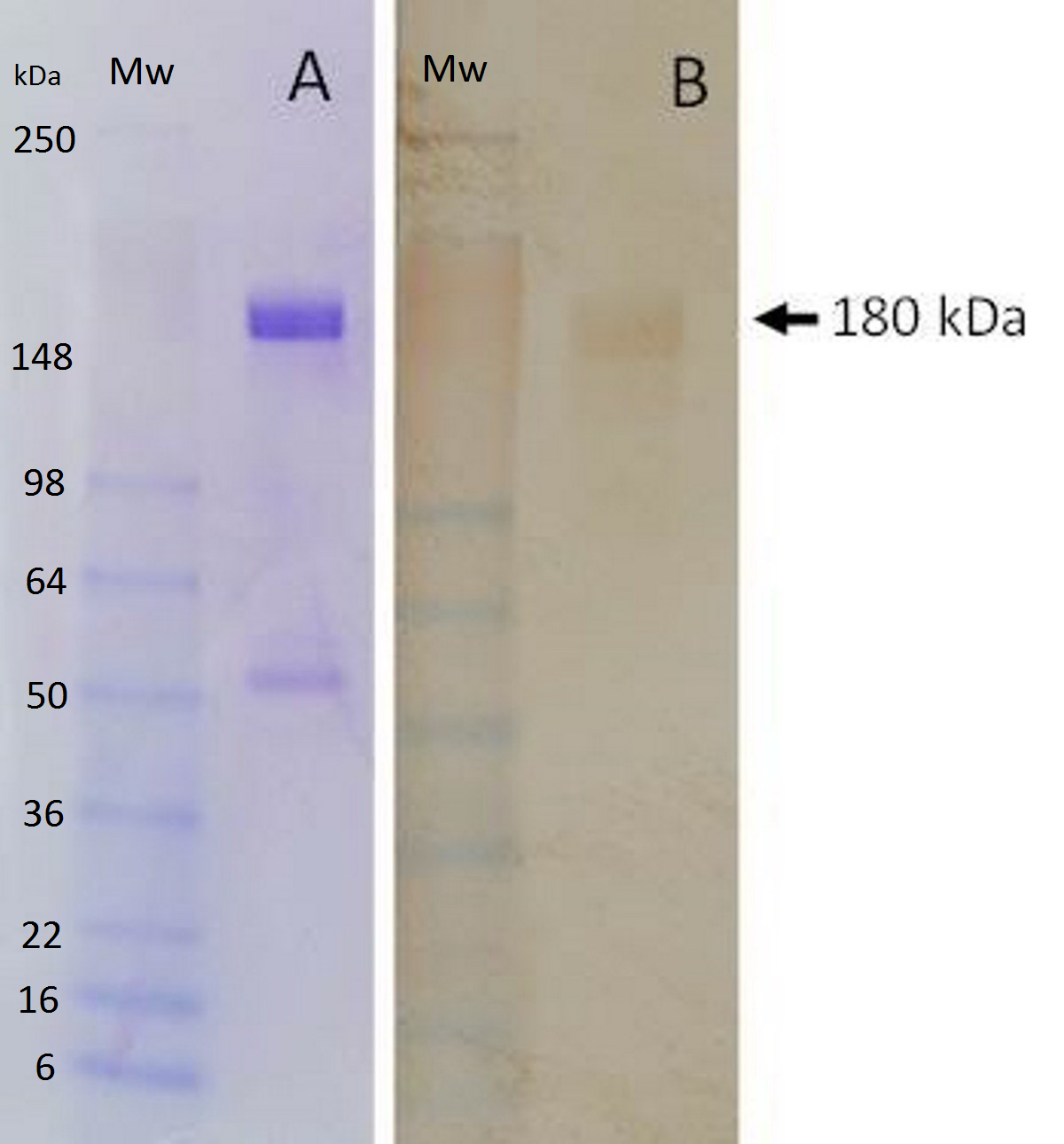

Supplement: S1 Fig — A: SDS-PAGE (under non-reducing conditions) of D-dimer antigen. B: Western blotting analysis of D-dimer antigen with a commercially available anti-D-dimer monoclonal antibody (HyTest Ltd.). Mw: Molecular weight marker—SeeBlue Plus2 Pre-stained Protein Standard (Invitrogen). (TIFF) [file pone.0212104.s002.tiff]
